# American Dental Association

**Published:** 1899-08

**Authors:** 


### EDITORIAL.

### American Dental Association.

The second annual meeting of the National Dental Association, re-organized, was held at Niagara Falls, N. Y., Aug. 1st to 4th, inclusive. A larger number was in attendance than ever before, either at this body or at the meetings of its predecessor. There were between 400 and 500 present. The meeting was a very good one indeed, and, under the conduct of its admirable president, did most excellent work. A number of very interesting papers were read and discussions had upon them, which we propose to present in the next two numbers of THE REGISTER. The papers in quality were above the ordinary, and the discussions in the main were by the most able men in the profession. Comparatively few of the papers were read, quite a number, however, were read by title and ordered to be published—quite a considerable number were not read even by title nor referred to the publication committee. If any criticism may be made, it is that more papers were written than could be read, which is always unfortunate. Persons who devote time and labor to the preparation of papers should have opportunity to present them, according to the original intent, otherwise there is disappointment and sometimes disaffection. This applies, however, only to persons who have been solicited to prepare such papers, and not to volunteers, though volunteer papers may oftentimes be used with very great advantage. It was unfortunate for the body that a hall to accommodate all the delegates could not be secured. The one that was used was not sufficient to accommodate more than one-half of those who desired to be present, the hall seating not over 225 persons. The temperature though not equal to that of last year was at times very oppressive, and the crowded hall increased this disability. Nearly all of the State Societies of the country were present by representatives. A large addition was made to the membership.

The Association under its new organization seems to work very well indeed, and great expectations are entertained for it in the future. In this all the members share.

Mid-summer has so often by its great heat seriously embarrassed the Association, that it seems a change of time for the meeting ought to be made, especially when the meeting is held in the south, or anywhere that the heat is liable to be great in mid-summer. In May or the latter part of September would be more favorable. We trust the Executive Committee will take the hall question and the time question into serious consideration.

